# Understanding and acting on the developmental origins of health and disease in Africa would improve health across generations

**DOI:** 10.1080/16549716.2017.1334985

**Published:** 2017-07-18

**Authors:** Shane A. Norris, Abdallah Daar, Dorairajan Balasubramanian, Peter Byass, Elizabeth Kimani-Murage, Andrew Macnab, Christoff Pauw, Atul Singhal, Chittaranjan Yajnik, James Akazili, Naomi Levitt, Jihene Maatoug, Nolwazi Mkhwanazi, Sophie E. Moore, Moffat Nyirenda, Juliet R. C. Pulliam, Tamsen Rochat, Rihlat Said-Mohamed, Soraya Seedat, Eugene Sobngwi, Mark Tomlinson, Elona Toska, Cari van Schalkwyk

**Affiliations:** ^a^ Stellenbosch Institute for Advanced Study (STIAS), Wallenberg Research Centre at Stellenbosch University, Stellenbosch, South Africa; ^b^ MRC/Wits Developmental Pathways for Health Research Unit, Department of Paediatrics, Faculty of Health Sciences, University of the Witwatersrand, Johannesburg, South Africa; ^c^ Dalla Lana School of Public Health and Department of Surgery, University of Toronto, Toronto, Canada; ^d^ L V Prasad Eye Institute, Hyderabad, India; ^e^ Department of Public Health and Clinical Medicine, Umeå University, Umea, Sweden; ^f^ African Population and Health Research Center, Kenya; ^g^ Department of Pediatrics, University of British Columbia, Vancouver, Canada; ^h^ Institute of Child Health, University College London, London, UK; ^i^ King Edward Memorial Hospital Research Centre, Pune, India; ^j^ INDEPTH Network, Ghana; ^k^ Department of Diabetic Medicine and Endocrinology, University of Cape Town, Cape Town, South Africa; ^l^ Department of Epidemiology, Hospital Farhat Hached, Sousse, Tunisia; ^m^ Department of Anthropology, University of the Witwatersrand, Johannesburg, South Africa; ^n^ Division of Women’s Health, King’s College London, London, UK; ^o^ College of Medicine, University of Malawi, Zomba, Malawi; ^p^ DST-NRF Centre of Excellence in Epidemiological Modelling and Analysis (SACEMA), University of Stellenbosch, Stellenbosch, South Africa; ^q^ Human and Social Development Research Programme, Human Sciences Research Council, Durban, South Africa; ^r^ Department of Psychiatry, Stellenbosch University, Stellenbosch, South Africa; ^s^ Department of Applied Epidemiology, University of Yaoundé, Yaounde, Cameroon; ^t^ Department of Psychology, Stellenbosch University, Stellenbosch, South Africa; ^u^ Department of Social Policy and Intervention, University of Oxford, Oxford, UK

**Keywords:** Africa, developmental origins of health and disease (DOHaD), non-communicable disease, life course epidemiology, policy, Sustainable Development Goals (SDGs)

## Abstract

Data from many high- and low- or middle-income countries have linked exposures during key developmental periods (in particular pregnancy and infancy) to later health and disease. Africa faces substantial challenges with persisting infectious disease and now burgeoning non-communicable disease.This paper opens the debate to the value of strengthening the developmental origins of health and disease (DOHaD) research focus in Africa to tackle critical public health challenges across the life-course. We argue that the application of DOHaD science in Africa to advance life-course prevention programmes can aid the achievement of the Sustainable Development Goals, and assist in improving health across generations. To increase DOHaD research and its application in Africa, we need to mobilise multisectoral partners, utilise existing data and expertise on the continent, and foster a new generation of young African scientists engrossed in DOHaD.

Developmental origins of health and disease (DOHaD) has become a globally recognised concept (see the DOHaD Cape Town manifesto) [1]. DOHaD is a multi-disciplinary field, exploring how environmental factors acting during early life (in particular the period of pregnancy and infancy) interact to change individual trajectories that may increase risk for health conditions in later life. The scientific evidence of DOHaD overwhelmingly demonstrates that the environment in which the embryo, fetus and child grows and develops influences both short-term (stunting risk; cognitive function) and longer-term health and wellbeing (non-communicable diseases [NCDs]; human capital) [2]. Consequently, the first 1000 days (from conception to second birthday) are critically important, but there is recognition that the pre-conception period and later years also matter. DOHaD science mandates a life-course approach, recognising that different needs emerge at various stages in life (such as pregnancy, infancy, childhood, adolescence and parenthood) [3], which form an intergenerational cycle (Figure 1). Investing in early-life interventions could effectively promote healthier trajectories lifelong and across generations.

**Figure 1.**
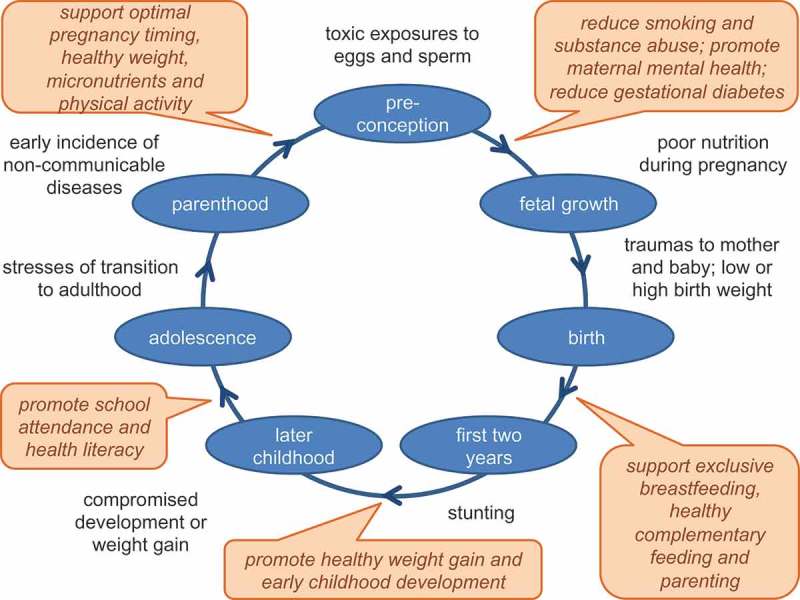
The DOHaD intergenerational cycle, annotated with examples of relevant stresses and exposures and indicating (in italic text boxes) examples of recommended interventions.

Africa has persisting burdens of malnutrition and infectious diseases (particularly HIV, TB and malaria). However, Africa now also faces a growing NCD encumbrance. Already by 2010, over two million deaths in Sub-Saharan Africa were due to NCDs and this was a 46% increase from 1990 [4]. In terms of DOHaD science, the combination of early life malnutrition (under-nutrition) coupled with excess weight gain in childhood, adolescence and adulthood accelerate increases in NCDs, particularly type-2 diabetes [5]. This epidemiological transition in Africa (increasing incidence of cardio-metabolic disease against a backdrop of maternal and child malnutrition such as anaemia and stunting), which is at varying stages of transition across the African continent [6], will be detrimental not only in health terms, but also for economic development due to escalating productivity losses and health care costs [7]. Shifting our attention to prevention would equate to millions of deaths averted and economic losses reduced [8]. DOHaD provides important insights for such a shift to prevention of NCDs in Africa and is closely aligned with many of the targets enshrined within the United Nations’ Sustainable Development Goals [9]. For example, ensuring gender equity will empower mothers to make better decisions for themselves and their children.

The recent Lancet Series on Early Childhood Development underscores the importance of ‘nurturing care’ and multi-sectoral health interventions in early childhood can bring extensive benefit to families and young children [10]. A number of evidence-based, cost-effective opportunities to improve maternal and infant health have been documented [11]. Some strategies for consideration include: optimising maternal and infant nutrition (nutrient supplementation), reducing foetal exposure to toxins (such as smoking and alcohol) and tackling antenatal and postnatal maternal mental health conditions (such as depression and anxiety). We believe that if these are applied systematically as part of national health strategies they will reduce the incidence and adverse effects of NCDs (obesity, type-2 diabetes, hypertension, coronary heart disease, chronic kidney disease, musculoskeletal disorders, some mental health conditions and a range of cancers) and improve human capital. A key question is how these proposed interventions could be successfully and sustainably implemented in Africa. Africa has had public health implementation success stories, for example: (1) Zambia’s Malaria Booster Project reduced malaria-linked cases and mortality by 31% and 37% respectively between 2006 and 2008; (2) HIV prevention efforts in many African countries resulted in observed behaviour change in young men and women and an increased use of condoms; and (3) The Southern Africa initiative has almost eliminated childhood mortality from measles in several African countries through effective vaccination campaigns (http://blogs.worldbank.org/africacan/african-successes-listing-the-success-stories). Learning from these, and other examples, could provide insight into how to implement NCD prevention interventions across the life-course; however, more research is still needed to add to the local evidence base. Science funders and institutions, like the African Academy of Sciences, could play a significant role in highlighting the gaps and supporting African scientists.

To further advance the successful implementation of DOHaD science, there is also a need for political vision, commitment and leadership at the highest level to encourage national dialogue within the African context. Multi-national efforts that may draw on the African Union could be particularly effective in facilitating dialogue and supporting scientific efforts. It is crucial that concerned players from all sectors come together to design and implement programmes aimed at improving current and future health through prevention.

Africa lacks a critical mass of scientists and researchers in many fields, and DOHaD is no exception. There is a need to attract and mentor young investigators to ensure that the future research necessary to advance DOHaD-related science can call on the necessary human resources. Also, the DOHaD field needs to inform and engage with the existing very high quality scientists on the continent whose research may be relevant. The DOHaD paradigm has only been explored in a few African countries, possibly due to limited longitudinal data from birth to adulthood [12]. This situation cannot be remedied in the short term, but there may be indirect methods that could be used on existing data (for example, data from African demographic surveillance sites that are part of the INDEPTH network; www.indepth-network.org), to assess the likely extent of DOHaD effects in African populations.

Africa is heterogeneous, so context matters. There are not only distinct differences between African countries, but often, within a country there are regional cultural differences. Therefore, health policies, including those to address DOHaD, should take this into account. For example, while prevention of stunting and promotion of linear growth clearly has long-term benefits for health and human capital [13], faster weight gain in infancy is also associated with a greater risk of obesity and hence NCDs [14]. Therefore, for one population the intervention may be more centred on promoting infant linear growth with less concern around faster weight gain, while in another population and context minimising excessive infant weight gain may be more relevant to offset risk in later life [15]. Also, as the levers to foster healthy nutritional practices and patterns of growth in infancy may vary due to cultural practices and context, more multi-site studies that take into account population and cultural variation within and between African countries could be particularly helpful to elucidate pathways to impact [16,17].

Africa’s mothers, fathers and families need to be empowered as critical agents for change in setting up healthier trajectories for their children (with knowledge from pre-conception, support during pregnancy, breastfeeding and child nutrition). This can only be achieved through a process of broad societal engagement, adopting DOHaD-informed practices as feasible, positive and lifelong options. Africa’s youth are the next generation of parents and are still able to modify their individual NCD trajectories. DOHaD-informed knowledge and health practices need to be integrated into school curricula and other youth-oriented arenas, using compelling messaging and novel means of engagement [18].

In conclusion, DOHaD principles in Africa need to move forward in a joint evidential and implementation-focused programme. The knowledge base for implementation is by no means complete but waiting to gather further intergenerational evidence is not an option as the potential risks to health and human capital are too high.

## Paper context

Africa faces multiple burdens (infectious and non-communicable diseases). From substantial evidence linking maternal and nutrition exposures that occur during pregnancy and infancy to adult health and disease, we propose a life-course framework to guide research and public health programmes in Africa to optimise health. The implementation of this framework could see African researchers contributing more to the field of developmental origins of health and disease, as well as offsetting such intergenerational challenges as stunting.
